# Influence of Baduanjin on cardiopulmonary function in long-term practitioners and beginners

**DOI:** 10.3389/fspor.2024.1440871

**Published:** 2024-11-18

**Authors:** Mengni Shi, Zhiwei Wu, Xin Zhou, Min Fang, Qingguang Zhu

**Affiliations:** ^1^ Institute of Tuina, Yueyang Hospital of Integrated Traditional Chinese and Western Medicine, Shanghai University of Traditional Chinese Medicine, Shanghai, China; ^2^ Biomechanics Laboratory, Institute of Tuina Research, Shanghai Research Institute of Traditional Chinese Medicine, Shanghai, China; ^3^ Department of Tuina, Shuguang Hospital, Shanghai University of Traditional Chinese Medicine, Shanghai, China

**Keywords:** Baduanjin, physical and mental exercise, exercise intensity, heart rate, oxygen consumption

## Abstract

**Objective:**

Baduanjin, a traditional Chinese exercise for health enhancement and chronic disease prevention, has been practiced for millennia. However, studies on the exercise intensity of Baduanjin are limited. Most existing studies focus on its general health benefits rather than quantifying its specific intensity levels. This study aims to measure and compare the exercise intensity indices of long-term Baduanjin practitioners and beginners, providing insights into its mechanisms for disease prevention and treatment and supporting the scientific formulation of clinical exercise prescriptions.

**Methods:**

Twenty healthy adults aged 35–45 years old and the mean BMI was 24.45 were recruited and divided into a beginner group (A group, no prior practice, 10 participants) and a skilled group (B group, practice duration ≥3 years, 10 participants). The Italian Cosmed/K5 wireless portable cardiopulmonary testing system was used to measure indicators during the practice.

**Results:**

Within-group analysis revealed statistically significant differences in oxygen consumption (VO_2_), oxygen consumption per kilogram of body weight (VO_2_/kg), metabolic equivalent (METs), heart rate (HR), oxygen pulse (VO_2_/HR), respiratory rate (RR), and minute ventilation (VE) between exercise and resting states in both the B and A groups (*P* < 0.001). In between-group comparisons, resting HR was significantly lower in the B group compared to the A group (*P* < 0.01). During Baduanjin practice, significant between-group differences were found in METs, HR (*P* < 0.01), and RR (*P* < 0.05), with the A group exhibiting higher values for METs, HR, and RR than the B group.

**Conclusion:**

Baduanjin positively impacts cardiovascular function and exercise performance, with long-term practitioners showing significantly better cardiovascular recovery and overall function.

## Introduction

Baduanjin, a traditional Chinese exercise (TCE), can be traced back to the Northern Song Dynasty (1). Characterized by smooth, graceful movements, it emphasizes fluidity, coordination, and balance, harmonizing qi and blood, yin and yang, and regulating the meridians (2). The practice comprises eight movements, beginning with a preparatory posture that aligns the body and breathing before transitioning into a series of gentle, symmetrical motions. These movements promote natural adaptation, inner peace, and tranquility (3). As a form of physical therapy, Baduanjin, effectively integrates breathing and body movements, fostering both physical and mental harmony, which makes it a powerful tool for preventive healthcare (4). TCEs are comprehensive mind-body exercises widely applied in the treatment of conditions such as hypertension (5). Numerous studies have reported significant positive effects on physical and physiological outcomes following TCE interventions (6). In particular, research has demonstrated that Baduanjin enhances physical, cognitive, and psychological health (7, 8).

This study focuses on Baduanjin because, unlike other TCEs, it emphasizes slow, gentle, and continuous movements. These qualities provide unique benefits in rehabilitation training and the management of chronic diseases. Additionally, Baduanjin is simple to learn, with low physical demands, making it accessible to individuals of various ages and fitness levels. Although extensive research exists on the health benefits of other traditional exercises, quantitative studies on Baduanjin, particularly regarding its exercise intensity and health impacts, are limited. In a modern society where work-related stress and sedentary lifestyles are increasingly common, many individuals seek exercises that can be practiced in limited spaces. Baduanjin is not limited by venue and it has shown positive effects on various postoperative conditions and diseases, including coronary heart disease, hypertension, type 2 diabetes, and stroke. Therefore, research on Baduanjin can provide scientifically grounded exercise recommendations for individuals facing these health challenges, helping to reduce work-related stress and improve their health (9–11).

A key factor in optimizing the benefits of any exercise is understanding its exercise intensity—one of the primary variables influencing exercise load and outcomes. Since individual fitness levels, health goals, and medical conditions vary, tailoring exercise intensity is essential for effective and personalized health management. Proper regulation of exercise intensity ensures safety, particularly for individuals with underlying medical conditions or physical frailty. Scientific quantification of exercise intensity enables better management of exercise load and plays a critical role in disease prevention and rehabilitation training. However, research on the exercise intensity of Baduanjin remains scarce. As such, studying the exercise intensity of Baduanjin is crucial, particularly for promoting personalized health management and developing evidence-based exercise prescriptions.

This study aims to utilize Cardiopulmonary Exercise Testing (CPET) to measure and evaluate the differences in exercise intensity between long-term practitioners and beginners during Baduanjin practice. CPET is a widely-used, comprehensive method for assessing cardiopulmonary function during exercise. By monitoring the responses of the heart, lungs, circulatory system, and muscles under progressively increasing exercise loads, it provides a detailed evaluation of both cardiopulmonary and metabolic systems. What distinguishes CPET is its ability to simultaneously measure multiple physiological parameters, including oxygen consumption (VO_2_), oxygen consumption per kilogram of body weight (VO_2_/kg), metabolic equivalent (METs), heart rate (HR), and more, allowing for precise assessment of cardiopulmonary function and exercise endurance. Extensive research has demonstrated CPET's broad applicability in clinical and exercise settings. For example, in patients with cardiovascular disease, CPET can effectively predict prognosis, assess cardiac function, and guide exercise prescriptions (12). CPET is also widely used to evaluate patients with chronic obstructive pulmonary disease (COPD), aiding in the determination of disease severity, lung function, and oxygen utilization efficiency (13). In sports medicine, research indicates that CPET is crucial for quantifying exercise loads, developing personalized training plans, and optimizing rehabilitation (14, 15). In this study, CPET serves as a distinctive assessment tool, providing quantitative evaluations of exercise intensity among Baduanjin practitioners. This comprehensive method enables accurate differentiation of the physiological responses between long-term practitioners and beginners.

We hypothesize that experienced practitioners will exhibit lower exercise intensity levels during practice compared to beginners, while demonstrating superior health outcomes. Furthermore, this study seeks to provide reference data to explore Baduanjin's preventive and therapeutic mechanisms and contribute to the scientific development of clinical exercise prescriptions.

## Methods

A small sample estimation was adopted based on the conservative principles and the capacity of the participants using G*Power software for power analysis, with efficiency as the estimation basis (*α* = 0.05, 1−β = 0.80, Cohen's d = 0.5) (16) and considering dropout rates. Ten healthy adults who have practiced Baduanjin for a long time and ten beginners were selected, forming the beginners group (A group) and the skilled group (B group). Inclusion Criteria: 1.Participants who have never practiced Baduanjin, aged between 35 and 45 years, regardless of gender. 2. The practice frequency for Baduanjin practitioners is at least once a week, following the video recommended by the General Administration of Sport of China, with a duration of no less than 12 min per session. These participants have been practicing consistently for over 3 years, are aged between 35 and 45 years, and are of any gender. Exclusion Criteria: (1) Individuals with limb injuries that may affect movement. (2) Individuals allergic to self-adhesive elastic bandages. (3) Individuals who have engaged in activities that could influence the results (such as weightlifting, swimming, running, badminton, etc.) within the past month.The participants voluntarily participated and signed informed consent forms. This study obtained approval from the Ethics Committee of Yueyang Hospital of Integrated Traditional Chinese and Western Medicine, Shanghai University of Traditional Chinese Medicine (approval no. 2023-192). Table 1 shows the biographic information of the subjects:

**Table 1:** Biographic information of the subjects in two groups (mean ± SD).

Indicators	A group (*n* = 10)	B group (*n* = 10)	*P* value
Gender	Male (*n* = 5); Female (*n* = 5)	Male (*n* = 5); Female (*n* = 5)	1
Age (years)	44.50 ± 4.0	42.90 ± 8.3	0.590
Height (cm)	166.30 ± 8.8	169.50 ± 7.0	0.382
Weight (kg)	68.10 ± 13.1	65.00 ± 12.8	0.598
Body mass index (BMI, kg/m^2^)	24.45 ± 3.1	22.45 ± 3.2	0.169

**Figure 1:**
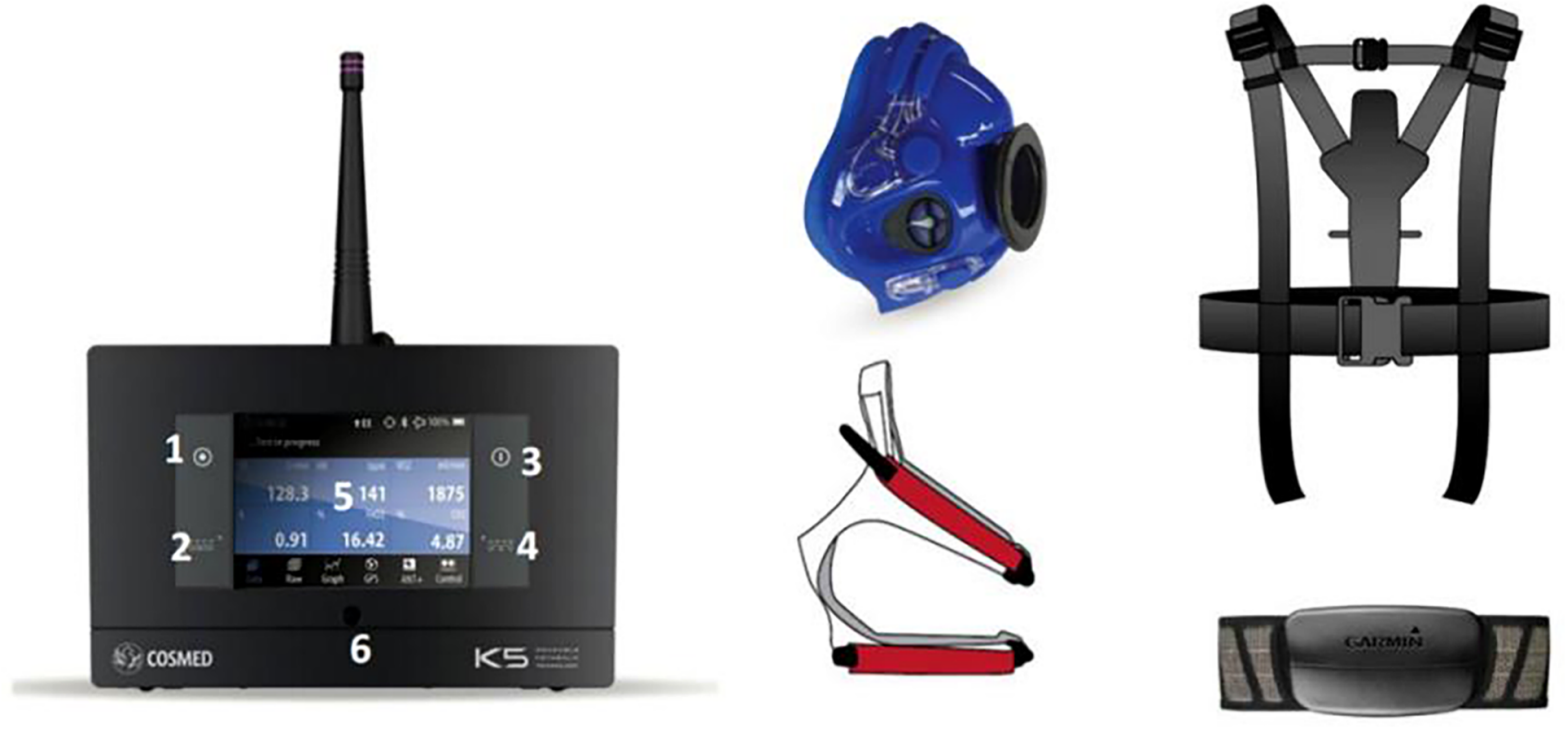
Cosmed/K5 exercise cardiopulmonary testing system.

CPET (Cardiopulmonary Exercise Testing) is a comprehensive testing method used to assess the functional status of the cardiopulmonary system during exercise. By monitoring the cardiopulmonary and metabolic responses under progressively increasing exercise loads, CPET provides a thorough evaluation of the heart, lungs, circulatory system, and the overall metabolic capacity of the muscles.This study utilised a wireless portable cardiopulmonary exercise testing (CPET) system (origin: Italy; brand/model: Cosmed/K5). The main unit is equipped with a built-in 3D Global Positioning System, with a horizontal accuracy of <2.5 m and a velocity accuracy of 0.1 m/s. The flowmeter is a bidirectional digital turbine type with permanent use capability, flow range of 0–16 L and ventilation range of 4–300 L/min. The accuracy is ±3%, and the flow resistance is <0.7 cm H_2_O/L/S@14 L/S. The oxygen analyser has an accuracy of ±0.02% and range of 0%–100%, whilst the carbon dioxide analyser has an accuracy of ±0.01% and range of 0%–12%. The mask has a flow range of 0–200 L/s and ventilation range of 10–2,500 L/min. Data transmission is achieved through Bluetooth telemetry technology. Figure 1 provides additional details.

Test Preparation: The participants were informed of basic precautions and trial information. Moreover, the participants refrained from engaging in vigorous high-intensity exercise within 24 h of the test day and maintained a fasting state. The experiment was conducted at the National Administration of Traditional Chinese Medicine Tuina Biomechanics Level III Laboratory, affiliated with Yueyang Integrated Traditional Chinese and Western Medicine Hospital, Shanghai University of Traditional Chinese Medicine. The testing environment was quiet with appropriate lighting, temperature maintained at 20°: Palatino Linotype>C–25°C and relative humidity at 60%. Three preliminary experiments were conducted before formal testing to minimise experimental errors.

**Figure 2:**
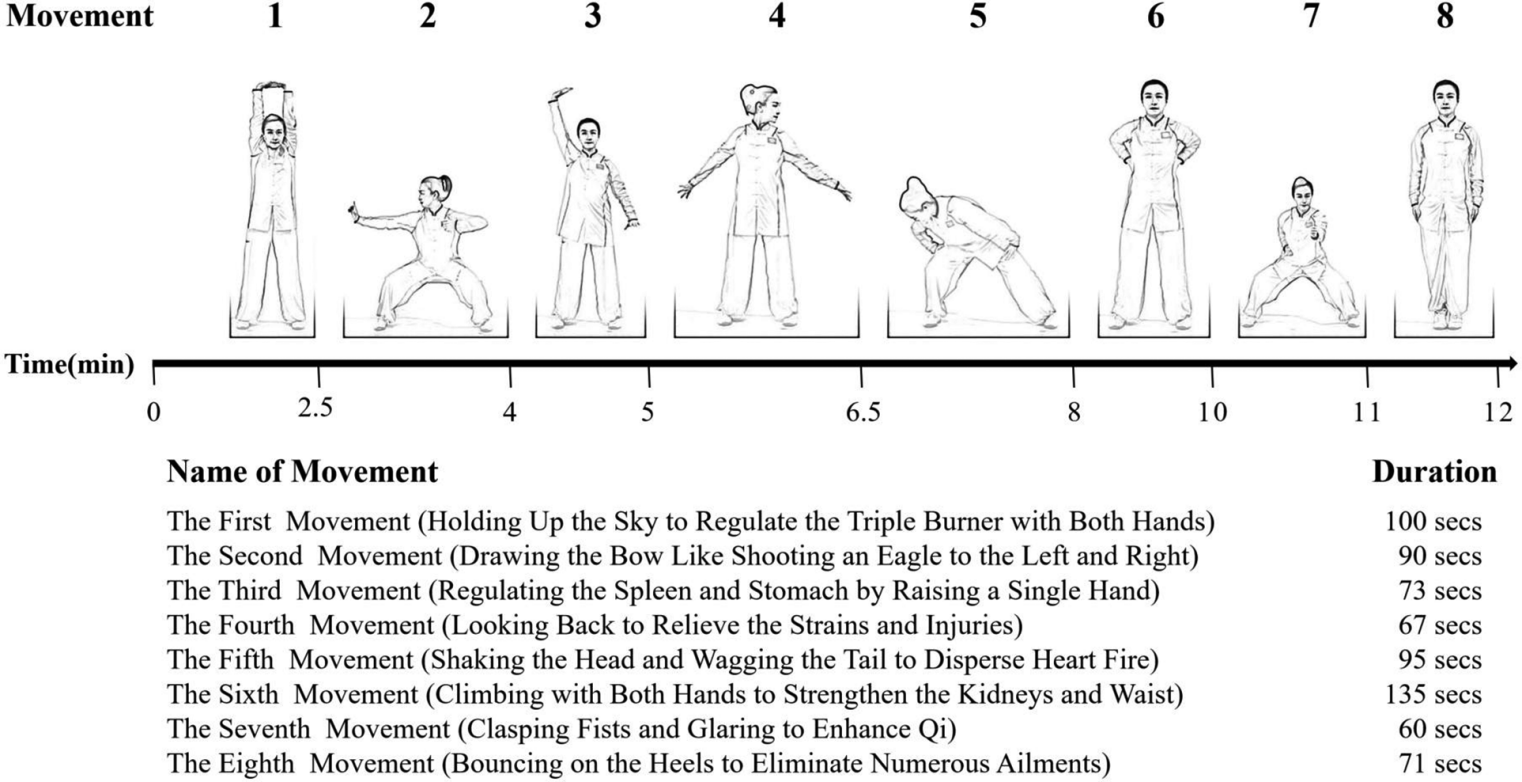
Figure of each movement of Baduanjin.

Testing Procedure: During testing, the participants wore loose clothing and the K5 cardiopulmonary telemetry device. The participants remained quiet and refrained from talking throughout the test. Prior to the formal start, the participants rested in a seated position for 15 min. Following the recommended video demonstration of Baduanjin by the General Administration of Sport of China (https://www.sport.gov.cn/n4/n24581921/n25423983/n25424052/c25433083/content.html), the participants ensured the continuity and accuracy of the test and completed the entire set of Baduanjin within the same time frame (12 min) (Figure 2).

The testing protocol utilised the CPET method, utilising a continuous ramp protocol. Real-time recording of the test results and relevant data was conducted. Data collection began in a resting state (≥3 min), followed by non-powered joint muscle warm-up exercises (e.g., chest expansion, waist rotation, leg lifting, etc., 5 min). Subsequently, the participants performed Baduanjin based on the recommended video by the General Administration of Sport of China (12 min). The recovery status was then recorded for ≥3 min post-exercise. During Baduanjin (within 12 min), the following variables were measured. Table 2 provides detailed data.

**Table 2:** Variables were measured.

Variables	Unit of measurement
– Oxygen consumption (VO2)	ml/min
– Oxygen consumption per kilogram of body weight (VO2/kg)	ml/min/kg
– Metabolic equivalent	METs
– Heart rate (HR)	beats per minute
– Oxygen pulse (VO2/HR)	ml/beat
– Respiratory rate (RR)	breaths per minute
– Minute ventilation (VE)	L/min
– Respiratory quotient (RQ)	–

The experimental data were analysed using SPSS 24.0 statistical software. The normality of the data was assessed using Q–Q plots and the Shapiro–Wilk test. Depending on the normality of the data, the paired-sample *t*-tests or Mann–Whitney *U*-tests were conducted for the within-group analysis (*α* = 0.05). Independent-sample *t*-tests or *U*-tests were used for the between-group analysis (*α* = 0.05). The data results are presented as mean ± standard deviation (SD), with significance set at *P* < 0.05 for the two-tailed tests.

## Results

### Exercise endurance and cardiac function indicators

The analysis of the cardiopulmonary exercise testing data for all participants revealed the following: The within-group comparisons during Baduanjin compared with the resting state showed significant differences in VO_2_, VO_2_/Kg, METs, HR and VO_2_/HR indicators for the B and A groups (*P* < 0.001). Immediate post-Baduanjin compared with the resting state, the B and A groups showed statistically significant differences in VO_2_ (*P* < 0.05), VO_2_/Kg (B group: *P* < 0.01, A group: *P* < 0.05), METs (B group: *P* < 0.01, A group: *P* < 0.001), HR(*P* < 0.01) and VO_2_/HR (no significant difference in B group, significant difference in A group, *P* < 0.001). In the three-minute post-Baduanjin compared with the resting state, no significant differences were observed in VO_2_, VO_2_/Kg and HR, whilst METs and VO_2_/HR showed no significant differences in the B group but significant differences in the A group (*P* < 0.01).

The between-group comparisons revealed statistical differences in HR at the resting state (*P* < 0.01), with the B group having a lower resting HR than the A group. During Baduanjin, METs and HR showed statistical differences (*P* < 0.01), with the A group exhibiting higher METs and HR than the B group. Immediate post-Baduanjin, METs and VO_2_/HR showed statistical differences (*P* < 0.01), with HR also showing differences (*P* < 0.05), all higher in the A group compared with the B group. Three-minute post-Baduanjin, METs and VO_2_/HR showed statistical differences (*P* < 0.05), with HR showing significant differences (*P* < 0.001), still higher in the A group compared with the B group. Table 3 provides detailed data.

**Table 3:** Exercise tolerance and cardiac function indicators (mean ± SD).

Indicators		Resting state	Between-group (*P* value)	During Baduanjin	Between-group (*P* value)	Immediate post -Baduanjin	Between-group (*P* value)	Three-minute post-Baduanjin	Between-group (*P* value)
VO_2_ (ml/min)	B	344.04 ± 94.26	0.314	745.07 ± 237.75***^a^	0.553	438.66 ± 124.77*^b^	0.599	361.38 ± 113.18	0.648
	A	300.92 ± 91.77		685.62 ± 200.84***^c^		400.78 ± 186.11*^d^		336.06 ± 130.34	
VO_2_/kg (ml/min/kg)	B	5.27 ± 0.98	0.101	11.33 ± 2.55***^a^	0.235	6.70 ± 1.15**^b^	0.383	5.47 ± 0.96	0.335
	A	4.43 ± 1.72		10.03 ± 2.18***^c^		5.89 ± 2.58*^d^		4.91 ± 1.52	
METs	B	1.41 ± 0.25	0.774	2.76 ± 0.35***^a^	0.007**	1.92 ± 0.33**^b^	0.002**	1.56 ± 0.27	0.020*
	A	1.37 ± 0.33		3.11 ± 0.14***^c^		2.38 ± 0.23***^d^		1.90 ± 0.32**^e^	
HR (bpm)	B	71.87 ± 5.91	0.001**	99.55 ± 6.10***^a^	0.003**	83.88 ± 9.53**^b^	0.012*	75.72 ± 7.13	0.000***
	A	84.30 ± 7.43		109.57 ± 6.87***^c^		93.40 ± 3.19**^d^		88.25 ± 4.22	
VO_2_/HR (ml/beat)	B	4.36 ± 1.33	0.682	9.38 ± 0.68***^a^	0.008**	5.07 ± 1.64	0.009**	4.25 ± 1.31	0.026*
	A	4.15 ± 0.86		8.21 ± 1.05***^c^		6.89 ± 1.08***^d^		5.47 ± 0.89**^e^	

^a^ During Baduanjin vs. resting state (B group).

^b^ Immediate post-Baduanjin vs. resting state (B group).

^c^ During Baduanjin vs. resting state (A group).

^d^ Immediate post-Baduanjin vs. resting state (A group).

^e^ Three-minute post-Baduanjin vs. resting state (A group).

* *P* < 0.05.

** *P* < 0.01.

*** *P* < 0.001.

### Pulmonary ventilation function indicators

The data analysis revealed that within each group, significant differences are observed in RR and VE between during Baduanjin and resting states for the B and A groups (*P* < 0.001), with RQ showing significant differences for the B group (*P* < 0.05). When comparing immediate post-Baduanjin with the resting state, the A group showed statistically significant differences in RR (*P* < 0.01), whilst the B and A groups exhibited significant differences in VE and RQ (*P* < 0.001 and *P* < 0.01, respectively). Additionally, when comparing three-minute post-Baduanjin with the resting state, VE showed significant differences (*P* < 0.05), with RQ showing significant differences for the B group (*P* < 0.01).

The between-group comparisons revealed statistically significant differences in RR during Baduanjin (*P* < 0.05), with the A group having higher RR than the B group. Immediate post-Baduanjin, RR (*P* < 0.01) and VE (*P* < 0.05) showed statistically significant differences, with the A group having higher RR and lower VE compared to the B group. Table 4 provides detailed data.

**Table 4:** Indicators of lung ventilation function (mean ± SD).

Indicators		Resting state	Between-group (*P* value)	During Baduanjin	Between-group (*P* value)	Immediate post-Baduanjin	Between-group (*P* value)	Three-minute post-Baduanjin	Between-group (*P* value)
RR (breaths per minute)	B	15.20 ± 3.53	0.181	20.44 ± 3.31***^a^	0.018*	14.87 ± 2.70	0.003**	14.43 ± 1.99	0.802
	A	12.91 ± 3.84		23.45 ± 1.60***^d^		19.26 ± 2.91**^e^		14.70 ± 2.75	
VE (L/min)	B	9.91 ± 2.63	0.236	19.44 ± 7.07***^a^	0.613	16.53 ± 4.50***^b^	0.042*	11.71 ± 3.69*^c^	0.417
	A	8.60 ± 2.15		18.06 ± 4.75***^d^		12.19 ± 4.36**^e^		10.41 ± 3.28*^f^	
RQ	B	0.91 ± 0.14	0.956	0.95 ± 0.13*^a^	0.825	1.07 ± 0.12***^b^	0.354	0.98 ± 0.10**^c^	0.445
	A	0.91 ± 0.18		0.94 ± 0.16		1.00 ± 0.19**^e^		0.93 ± 0.16	

^a^ During Baduanjin vs. resting state (B group).

^b^ Immediate post-Baduanjin vs. resting state (B group).

^c^ Three-minute post-Baduanjin vs. resting state (B group).

^d^ During Baduanjin vs. resting state (A group).

^e^ Immediate post-Baduanjin vs. resting state (A group).

^f^ Three-minute post-Baduanjin vs. resting state (A group).

^*^ *P* < 0.05.

^**^ *P* < 0.01.

^***^ *P* < 0.001.

## Discussion

CPET provides a comprehensive assessment of the physiological responses to exercise, evaluating the pulmonary, cardiovascular, muscular and cellular oxidative systems (17, 18). This mechanism helps in determining highly personalised training intensity zones and structured exercise plans (19). This study compared the exercise intensity indicators during Baduanjin practice between the B and the A groups and scientifically quantitatively evaluated the cardiopulmonary function and metabolic status during Baduanjin.

Monitoring revealed significant changes in exercise endurance, cardiac function, and pulmonary ventilation function indicators. During Baduanjin practice, the B group's average VO_2_ reached 745.07 ml/min, with VO_2_/Kg at 11.33 ml/min/Kg and VO_2_/HR at 9.38 ml/beat, all of which were significantly higher than those observed in the A group. Notably, heart rate (HR) measurements indicated that the B group had a resting HR that was considerably lower than that of the A group. While both groups experienced an increase in HR following exercise, the B group maintained a lower HR overall, suggesting that Baduanjin effectively improves autonomic nervous system function and enhances its coordination. This improvement is indicative of increased vagal nerve tension, ultimately benefiting cardiac function (20). The slower HR in the B group highlights that prolonged Baduanjin practice can lead to favorable adaptations within the cardiovascular system, resulting in a new state characterized by higher efficiency. This finding aligns with previous studies that have reported similar benefits from mind-body exercises, such as Tai Chi and Qigong, which have also been shown to enhance cardiovascular health and improve autonomic regulation. Comparatively, our results underscore the specific advantages of Baduanjin in fostering endurance and cardiac efficiency, supporting its integration into regular exercise regimens for health promotion.

Both groups demonstrated significant increases in respiratory rate (RR) and ventilation (VE), with the B group achieving an average RR of 20.44 breaths/min and VE of 19.44 L/min, while the A group recorded an average RR of 23.45 breaths/min and VE of 18.06 L/min. The increase in VE during respiration is primarily attributed to a rise in tidal volume, which refers to the amount of air inhaled or exhaled during a single breath at rest. This deeper breathing associated with Baduanjin strengthens respiratory muscles and enhances the elasticity of the chest and lungs, leading to improved lung function. These changes indicate that Baduanjin effectively mobilizes bodily functions, with aerobic exercise performance being particularly pronounced in the B group. Classifying Baduanjin as low-intensity exercise for the B group and moderate-to-low-intensity exercise for the A group, according to the Centers for Disease Control and Prevention (CDC) and the American College of Sports Medicine (ACSM) (21), underscores its accessibility as a form of physical activity. This classification suggests that prolonged Baduanjin practice enhances exercise tolerance in the B group, with the lower exercise intensity indicating a more efficient use of energy. Comparatively, similar studies have shown that mind-body practices like Tai Chi and Qigong also lead to improvements in respiratory function and exercise performance, supporting the notion that such exercises can significantly enhance aerobic capacity and respiratory efficiency. Our findings reinforce the importance of integrating Baduanjin into regular exercise routines, particularly for individuals seeking a low-impact yet effective method to enhance their respiratory health and overall physical performance.

In comparing the recovery status of the two groups immediately and 3 min after Baduanjin, significant differences were observed in exercise endurance and cardiac function indicators, including heart rate (HR), metabolic equivalents (METs), and VO_2_ per heart rate (VO_2_/HR). The B group exhibited a more rapid recovery, with their values returning closer to resting state levels. This suggests that long-term practice of Baduanjin enhances the body's ability to recover after exercise.Additionally, pulmonary ventilation function indicators, such as respiratory rate (RR) and ventilation (VE), showed significant differences between the groups. The B group's RR recovered more quickly immediately post-exercise, indicating more efficient respiratory recovery. This suggests that Baduanjin, when practiced over a longer period, improves not only cardiovascular recovery but also respiratory function. When comparing these findings to similar studies on mind-body exercises like Tai Chi and Qigong, the results align with the evidence that such exercises improve cardiovascular recovery and autonomic regulation. Prior research has shown that long-term engagement in mind-body practices increases parasympathetic activity, leading to better heart rate variability (HRV) and faster recovery from physical exertion. Our findings further emphasize the unique benefits of Baduanjin in this regard, particularly in enhancing recovery capacity. Compared to other exercises, Baduanjin offers a more moderate, accessible form of training that improves both cardiac and pulmonary recovery, reinforcing its role in health promotion and rehabilitation programs.

Baduanjin is a unique fitness regimen in China, which promotes flexibility, strengthens bones and muscles and enhances vitality (22). This exercise is believed in traditional medicine to regulate the breath, strengthen the body and coordinate the internal organs. The gentle and slow movements of Baduanjin can fully relax and activate the body's self-regulation function (23). Studies have also shown that Baduanjin has a beneficial effect on individuals’ physical and mental health (4–9). With the development of society and the improvement of living standards, health issues have received widespread attention, and people are seeking suitable ways to exercise to improve their health. However, issues, such as different populations, work schedules and locations, persist. Baduanjin is simple and easy to perform, and its eight movements provide the health benefits needed by the general population, without requiring specific exercise venues, equipment or time, meeting the requirements of most people. Regular exercise is essential for maintaining lasting and vigorous vitality, exercise is considered a remedy for various diseases (24), and increasing physical exercise can fully utilise the positive effects of exercise on disease prevention and health promotion. Baduanjin is a relatively safe exercise for practitioners, and the intensity and volume of exercise can be determined based on the quantitative data obtained in this study, with appropriate training volume being the most basic factor to ensure fitness effects.

## Limitations

The research findings can provide scientific data support for the formulation of clinical exercise prescriptions.The limitations of this study include the relatively small sample size and the restriction of the participant age range to 35–45 years. Future research should increase the sample size, expand the age range of participants, and conduct long-term follow-up studies for more comprehensive investigation.

## Conclusion

Baduanjin effectively mobilizes bodily functions, with a particular positive impact on cardiovascular function and exercise performance. Long-term practitioners exhibit significantly better cardiovascular recovery and overall function compared to non-practitioners, making Baduanjin a highly recommended daily exercise for improving heart health and enhancing physical endurance.

## Data Availability

The original contributions presented in the study are included in the article/Supplementary Material, further inquiries can be directed to the corresponding author.
